# Exploring the Phytochemical Composition of Bitter Melon Fruit and Molecular Responses to Its Extract in U87 MG Glioblastoma Cells

**DOI:** 10.3390/molecules31203714

**Published:** 2026-10-16

**Authors:** Chinreddy Subramanyam Reddy, Dilip Nagisetty, Aishwarya Gaikwad, Nicole Tendayi Mashozhera, Virender Kumar, Gwonjin Lee, Robert T. Harris, Gerald R. Hankins, Umesh K. Reddy

**Affiliations:** Department of Biology, Gus R. Douglass Institute, West Virginia State University, Institute, WV 25112, USA; subramanyam.chinreddy@wvstateu.edu (C.S.R.); dilip.nagisetty@wvstateu.edu (D.N.); gaikwadaishwarya23@gmail.com (A.G.); nmashozhera@wvstateu.edu (N.T.M.); virender.kuma@wvstateu.edu (V.K.); gwonjin.lee@wvstateu.edu (G.L.); harrisro@wvstateu.edu (R.T.H.); ghankins@wvstateu.edu (G.R.H.)

**Keywords:** bitter melon, anticancer, transcriptome data, cell viability

## Abstract

Glioblastoma (GBM) is a highly aggressive type of brain cancer for which only a limited number of therapeutic options are available, highlighting the need to use multi-target strategies that interfere with the mechanisms by which the tumor survives and adapts to stress. In the present study, we investigated the antiproliferative and transcriptomic effects of bitter melon (*Momordica charantia*) extract (BME) in U87 MG glioblastoma cells. GC-MS analysis of bitter melon fruit revealed several bioactive metabolites known to have anticancer properties, including stigmasterol, β-sitosterol, caffeic acid, ferulic acid, and other fatty and organic acids, indicating that the extract has a chemically diverse composition that may explain its biological effects. BME markedly inhibited U87 MG cell proliferation in a dose- and time-dependent manner, with an IC_50_ of 659 µg/mL. Transcriptome analysis identified 908 differentially expressed genes, 380 upregulated and 528 downregulated. Functional and pathway analyses demonstrated enrichment in apoptosis, cellular senescence, ferroptosis, p53 signaling, and cellular stress responses, together with the suppression of PI3K–AKT, TNF, TGF-β, and cytokine–cytokine receptor signaling. The findings show that BME’s growth-inhibiting effect results from coordinated activation of cell-death and stress-response pathways along with reduced pro-tumor survival signaling. Combining metabolite profiling with transcriptomic analysis provides a molecular basis for identifying additional compounds in BME that are responsible for its antiproliferative action in glioblastoma and for verifying their effects.

## 1. Introduction

Glioblastoma is the most frequent and most aggressive of the primary brain tumors occurring in adults. It grows quickly, spreads into surrounding brain tissue and generally exhibits necrosis and neovascularization [1,2]. The standard course of treatment starts with a biopsy followed by surgical removal, with the amount of tumor that can be removed being a major influence on the patient’s prognosis [3]. The purpose of surgery is to remove as much of the tumor as possible without losing neurological function [4]. After surgery, patients usually receive chemoradiotherapy, most commonly involving temozolomide (TMZ). Nevertheless, the prognosis is still very bad, with the number of patients in the United States who survive for five years being only 5 to 10% [5].

The effectiveness of existing glioblastoma treatments is limited by a number of factors, such as incomplete removal of the tumor, genetic heterogeneity, the blood–brain barrier, and an immunosuppressive tumor microenvironment [6]. Because of these problems, the treatment efficiency is reduced, and tumor recurrence is frequent, which in turn hinders the development of therapies that provide long-term relief [5,7]. Drug resistance, which is caused by cellular heterogeneity and particular molecular characteristics, represents a major barrier [8]. Consequently, conventional chemotherapy rarely leads to lasting remission and frequently results in serious side effects since it damages normal tissue [9].

Looking at the various compounds derived from plants that have anticancer properties provides a promising method of reducing the side effects of chemotherapy and improving patient outcomes [10,11]. *Momordica charantia*, commonly known as bitter melon, is a tropical plant belonging to the Cucurbitaceae family and has been shown to have anticancer activity. This effect is primarily caused by cucurbitane-type triterpenoids and their glycosides, the same compounds that are responsible for the bitterness of the fruit. The amounts and type of these compounds vary according to the variety of the plant, its place of origin, and the time at which it is harvested [12].

Preclinical studies have demonstrated that extracts of bitter melon and their active ingredients exhibit anticancer effects against a variety of cancers [13]. On this basis, we first investigated the metabolite profile of an extract from *Momordica charantia* fruit. As a result, several bioactive compounds were identified, including some that have already been reported to have antiproliferative and pro-apoptotic effects in various cancer models. In order to better understand the molecular mechanisms involved in glioblastoma, we then looked at the effect of the bitter melon extract on gene expression in U87 MG cells, with a particular focus on the pathways concerned with cell proliferation, survival, stress response, and programmed cell death.

## 2. Results

### 2.1. Metabolite Profiling and Identification of Bioactive Compounds from Bitter Melon Fruit

The analysis by metabolomic profiling revealed that there were 107 metabolites in the bitter melon fruit (Data S1). The metabolites identified were grouped into different main categories according to their chemical properties and metabolic functions, such as sugars and oligosaccharides, sugar acids and lactones, sugar alcohols and polyols, phosphorylated carbohydrates, amino acids and their derivatives, amines and polyamines, organic acids, fatty acids and lipid-related metabolites, phenolic and aromatic acids, sterols, vitamins and antioxidants, nucleosides, and other miscellaneous metabolites (Table 1). There was a large variation in the relative abundance of the individual metabolites, which shows that the metabolic profile is chemically diverse.

Several of the identified metabolites have previously been reported to exhibit antiproliferative, pro-apoptotic, or other biological activities related to cancer. The metabolites that stand out are stigmasterol, β-sitosterol, caffeic acid, ferulic acid, α-linolenic acid, lauric acid, stearic acid, palmitic acid, azelaic acid, ascorbic acid, and dehydroascorbic acid. Table 2 summarizes the relative abundances of selected metabolites, their reported anticancer activities, the cancer models investigated, and the proposed molecular mechanisms.

These metabolites are implicated in mechanisms including modulation of oxidative stress, induction of mitochondrial dysfunction, initiation of apoptosis, regulation of the cell cycle, and alteration of cancer-associated signaling pathways.

### 2.2. BME Treatment Reduces U87 MG Glioblastoma Cell Viability in a Time- and Dose-Dependent Manner

**Figure 1:**
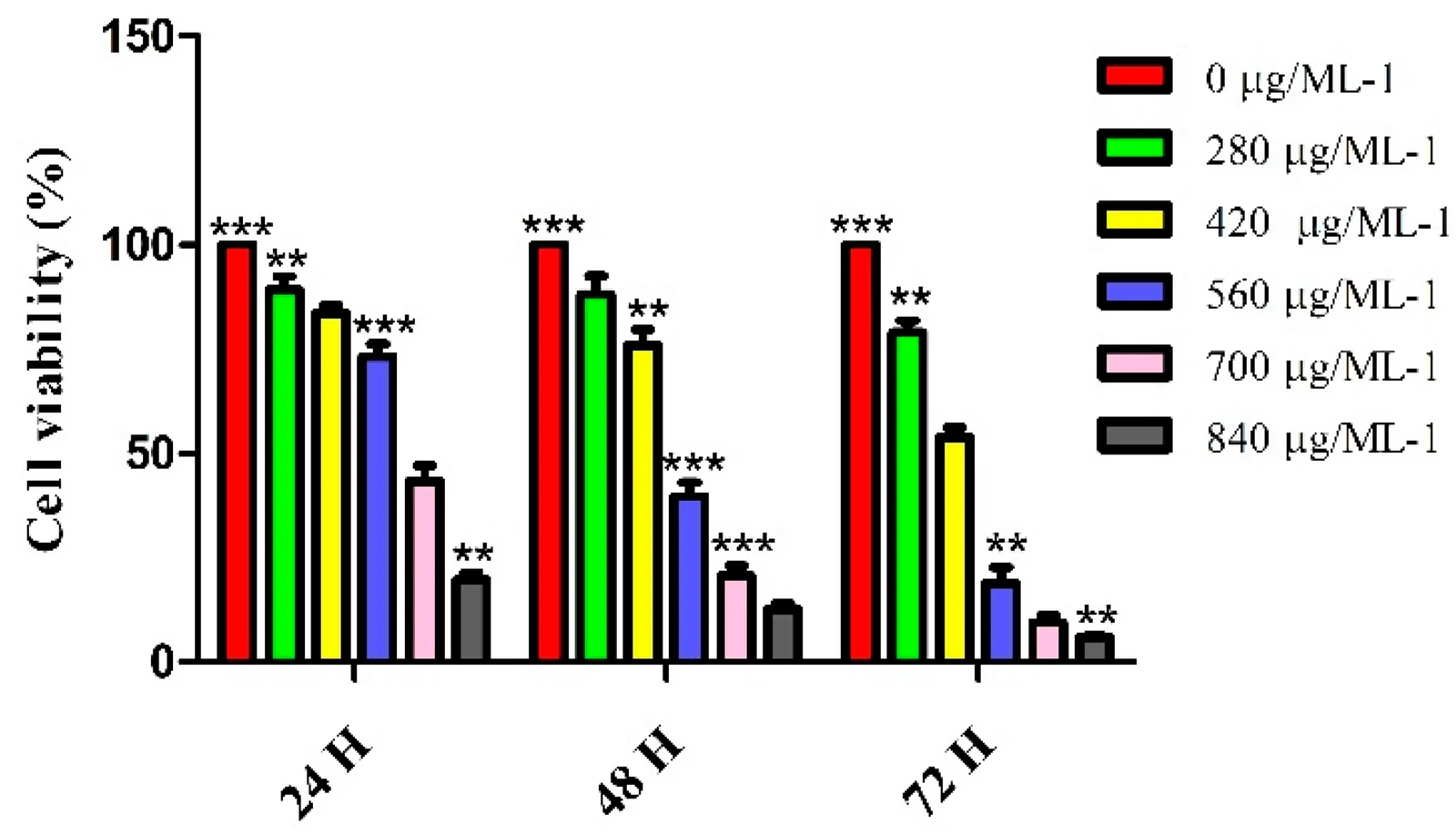
**Effect of bitter melon fruit extract on U87 MG glioblastoma cell viability.** U87 MG cells were treated with increasing concentrations of bitter melon extract (BME) for 24, 48, and 72 h. Vehicle-control cells received culture medium containing an equivalent concentration of ethanol (<0.1% *v*/*v*) and were normalized to 100% viability. Cell viability was assessed using the CCK-8 assay. Data are presented as mean ± SD (*n* = 6). Statistical significance was determined by two-way ANOVA, with *p* < 0.05, ** *p* < 0.01, and *** *p* < 0.001 compared with the vehicle control.

We tested the effects of bitter melon extract (BME) on U87 MG glioblastoma cells using a CCK-8 assay. BME reduced cell viability depending on dose and exposure time (Figure 1). At 24 h, concentrations between 280 and 560 µg/mL did not significantly affect cell viability, but by 48 h, 560 µg/mL began to reduce cell viability. At 72 h, even 420 µg/mL showed clear effects. This shows that BME’s cytotoxic effects increase with higher doses and longer exposure. The IC_50_ was 659 µg/mL (Figure S1), which we used for later experiments.

### 2.3. BME Treatment Resulted in Differential Gene Expression in U87cells

**Figure 2:**
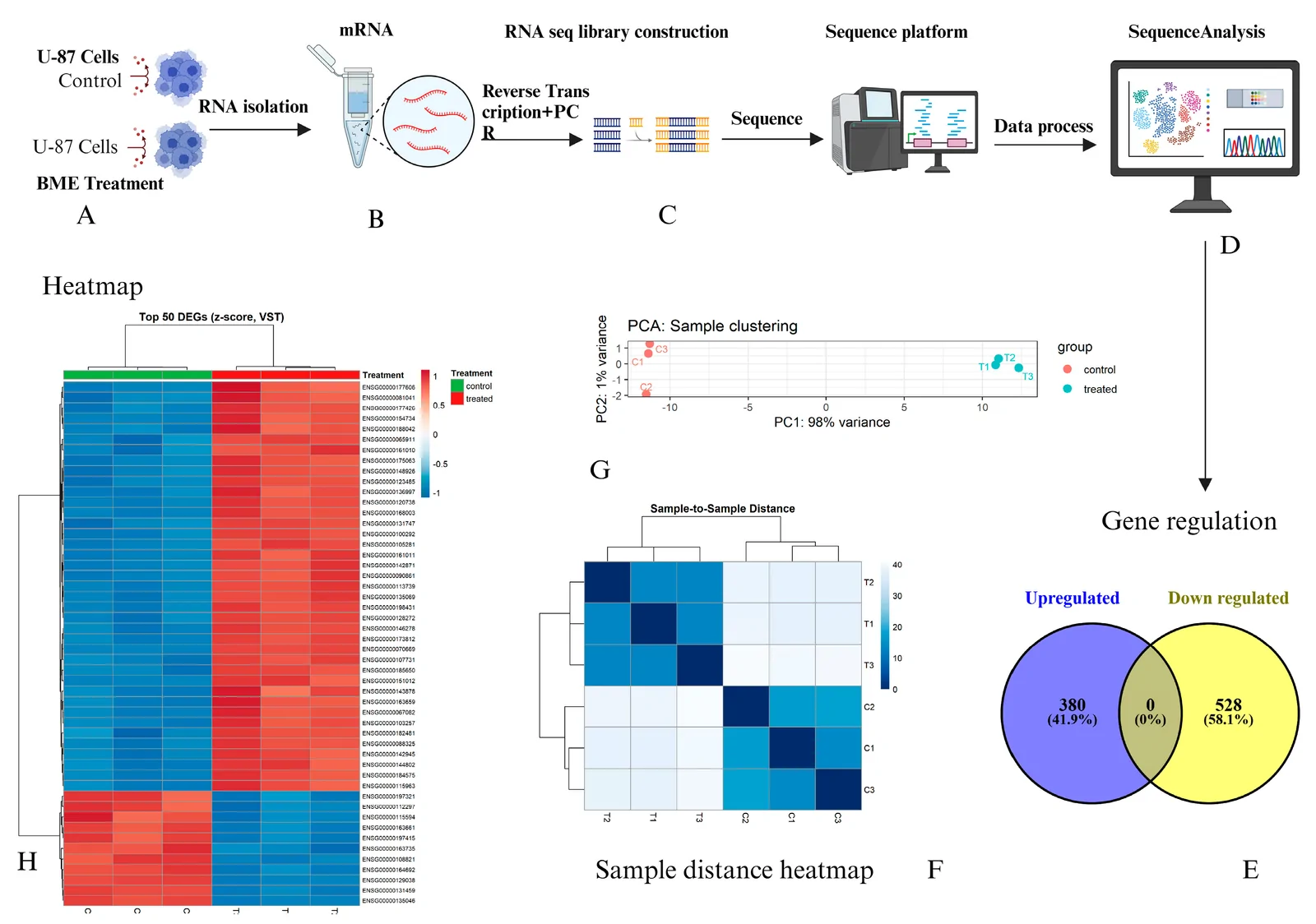
**Experimental workflow and transcriptomic profiling of bitter melon extract (BME)-treated U-87 cells.** (**A**) U-87 cells were divided into control and BME-treated groups, followed by total RNA extraction. (**B**) Replicate RNA samples were subjected to reverse transcription and RNA-seq library preparation. (**C**) Prepared libraries were sequenced using a high-throughput sequencing platform. (**D**) Sequencing data were processed and subjected to transcriptomic and bioinformatic analyses. (**E**) Differential gene expression analysis identified 380 upregulated (41.9%) and 528 downregulated (58.1%) genes, with no overlap between the two groups. (**F**) Sample-distance heatmap illustrating the similarity and separation among biological replicates. (**G**) Principal component analysis (PCA) showing distinct clustering of control and BME-treated samples based on their global gene expression profiles. (**H**) Hierarchical clustering heatmap displaying differential gene expression patterns between control and BME-treated U-87 cells. Together, these analyses demonstrate distinct transcriptomic alterations in U-87 cells following BME treatment.

RNA sequencing produced 332,999,712 raw reads. After removing low-quality sequences (Phred score below 30) and adapters, 321,194,991 high-quality reads remained. Sample-level quality was evaluated through PCA and sample-to-sample distance heatmaps based on variance-stabilized counts. The PCA plot (Figure 2G) shows tight clustering among biological replicates within each condition and clear separation between control and treated samples along the first principal component, which explains 98% of the variance. This indicates high reproducibility and strong condition-specific expression patterns.

Additionally, the sample-to-sample distance heatmap further confirms replicate consistency (Figure 2H). Hierarchical clustering grouped samples strictly by biological condition, with minimal within-group distances and no evidence of batch-related separation.

Dispersion estimates and Cook’s distance diagnostics from DESeq2 revealed no outlier samples (Figure S2). So, these results demonstrate that the count data were suitable for differential expression analysis and that biological replicates provided sufficient statistical power for detecting meaningful transcriptional changes. A total of 94% of the sequencing reads were successfully aligned to the human reference genome (GRCh38.p13). We compared gene expression between control and BME-treated samples (659 µg/mL) using DESeq2 in RStudio (version 3.0). Genes with a log2 fold change of at least 1 or at most −1 and a false discovery rate of 0.05 or less were considered significantly different. In total, 908 genes were significantly different, with 380 upregulated (Data S2) and 528 downregulated (Data S3). Venn diagrams summarize the upregulated and downregulated sets, and a heatmap displays their expression patterns (Figure 2).

### 2.4. KEGG Pathway and Gene Ontology Enrichment Analysis

**Figure 3:**
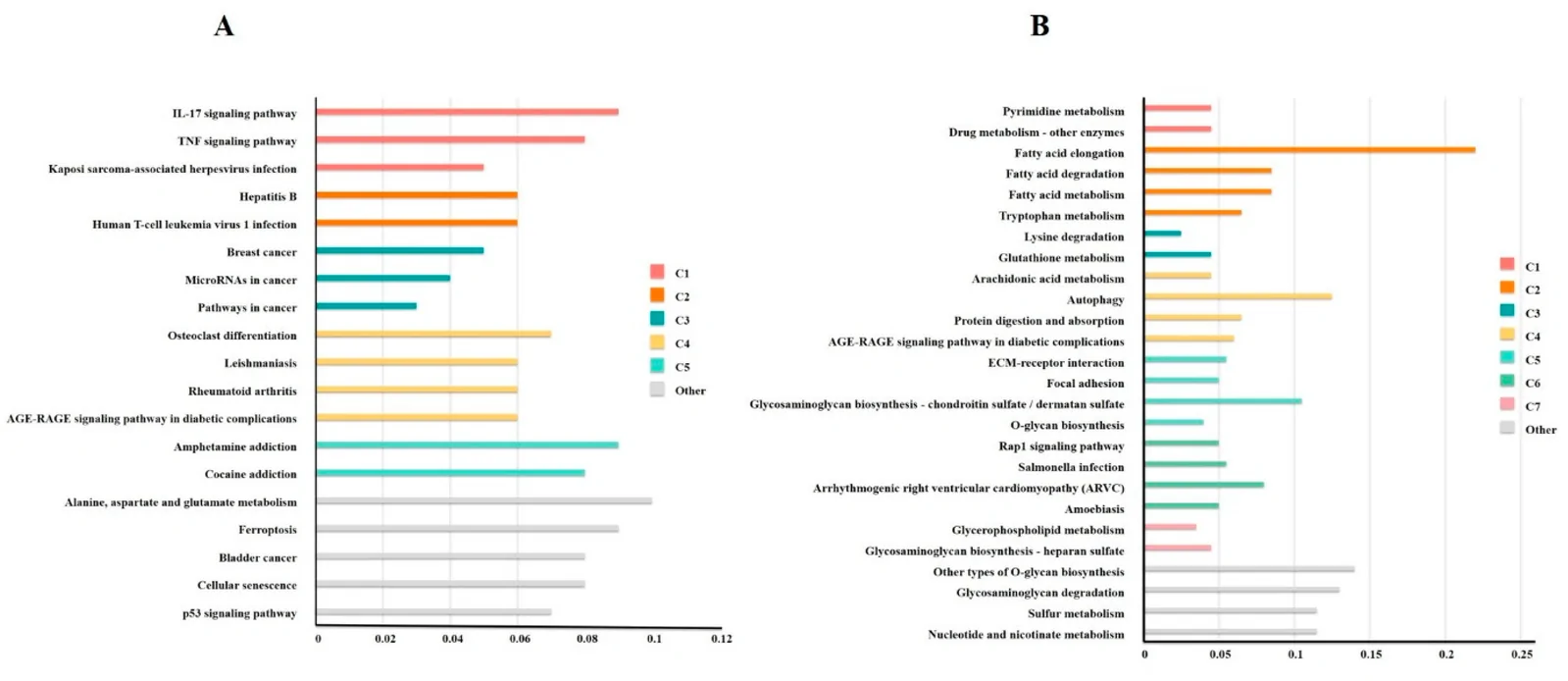
KEGG pathway enrichment analysis of differentially expressed genes (DEGs) in bitter melon extract (BME)-treated U87 MG glioblastoma cells. (**A**) KEGG pathway enrichment analysis of upregulated DEGs. (**B**) KEGG pathway enrichment analysis of downregulated DEGs. The horizontal bars represent the enrichment ratios of the corresponding KEGG pathways, with higher values indicating greater enrichment. Pathways were grouped into seven distinct clusters (C1–C7) using a clustering algorithm, with each cluster represented by a different color. Pathways not assigned to these clusters are categorized as “Other”. The analysis highlights the functional distribution of DEGs across pathways involved in inflammatory signaling, immune responses, cellular metabolism, and other biological processes following BME treatment.

We used KEGG pathway enrichment analysis to study the significant genes affected by BME. This helped us identify key biochemical, metabolic, and signaling pathways involved in BME’s effects on U87 glioblastoma cells. With a *p*-value below 0.05 and a minimum pathway size of 15, we found that the commonly upregulated genes were enriched in 85 pathways. Figure 3 shows the top 20 up- and down-regulated pathways. These upregulated genes were involved in MAPK signaling, cellular senescence, transcriptional misregulation in cancer, and the FoxO signaling pathway (Data S4). Downregulated pathways included metabolic pathways, cytokine–cytokine receptor interaction, NOD-like receptor signaling, fatty acid biosynthesis, and TNF and TGF-beta signaling. More data are available in the Supplementary Materials (Data S5).

We performed Gene Ontology (GO) enrichment analysis on the significant genes affected by BME to understand their functions. We grouped the enriched GO terms into biological process, cellular component, and molecular function. For upregulated genes, the most enriched biological processes were related to metabolic regulation, cellular processes, and cell-cycle control. These genes were mainly found in the nucleoplasm, chromosomes, nuclear lumen, chromatin, organelle lumen, and transcription regulator complexes. Their main molecular functions included DNA binding and transcription regulation. More details are in the Supplementary Data (Data S6). Downregulated genes were enriched in processes like response to peptides and cytokines, cell communication, and signal transduction. These genes were mostly found in the cytoplasm, extracellular region, cell edge, and membrane, with main functions in catalysis and cytokine binding. The top ten enriched GO terms for each category are in Supplementary Data (Data S7).

### 2.5. Transcriptomic Analysis Reveals a Complex Gene Regulatory Network Underlying BME Treatment Response

**Figure 4:**
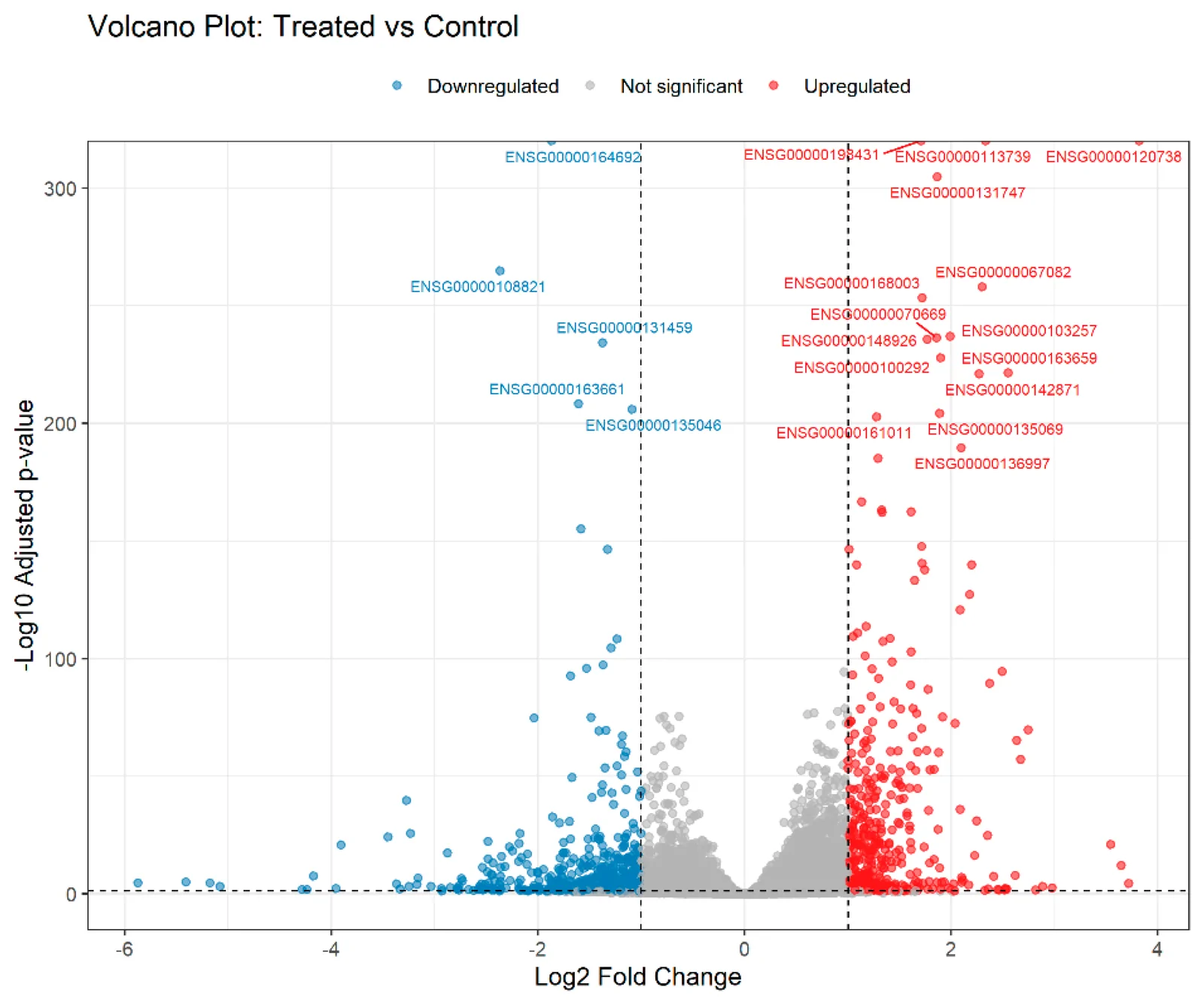
**Volcano plot of DEGs in BME-treated versus control U-87 cells.** The volcano plot illustrates changes in gene expression following bitter melon extract (BME) treatment. The x-axis represents log_2_ fold change (Log_2_FC), and the y-axis represents the −log_10_ adjusted *p*-value, indicating statistical significance. Red dots represent significantly upregulated genes, blue dots represent significantly downregulated genes, and gray dots indicate genes that did not meet the significance criteria. Dashed vertical lines indicate the fold-change thresholds. Selected highly significant DEGs are labeled with their Ensembl gene identifiers.

We used RNA sequencing to study how BME affects gene expression in U87 MG cells. Principal component analysis showed that the samples were clearly separated (Figure 2E). Treating cells with 659 μg/mL BME changed the expression of 908 genes, with 380 upregulated and 528 downregulated. Key upregulated pathways included MAPK signaling, cellular senescence, apoptosis, ferroptosis, TNF, FoxO, and p53 signaling. Downregulated pathways included metabolism, cytokine–cytokine receptor interaction, TNF, and PI3K-Akt signaling. More details are in the Supplementary Data (Data S2). Volcano plot analysis revealed distinct gene expression changes following BME treatment. The upregulated and downregulated genes were clearly separated based on their log_2_ fold change and adjusted *p*-values, while genes that did not meet the significance criteria remained unchanged. We identified several highly significant DEGs, indicating substantial transcriptomic alterations in response to BME treatment (Figure 4).

## 3. Discussion

Interest in using natural compounds to treat cancer has grown because they may reduce the side effects of standard chemotherapy. Bioactive plant chemicals like curcumin, capsaicin [1,9], and extracts from plants such as watermelon rind [10] and bitter melon [25] have been shown to slow cancer cell growth and affect key pathways in tumor development. This suggests that natural products could be safer and effective alternatives or additions to cancer therapy. Earlier studies found that bitter melon extract has strong anticancer effects in several cancer cell types, including breast cancer (MCF) [25,26,27], AGS gastric adenocarcinoma, HCT-116 colorectal carcinoma, and CL1-0 lung adenocarcinoma cells [28]. However, the global molecular mechanisms behind these effects remain poorly understood. Our study aims to clarify how bitter melon extract changes gene expression across the genome.

**Figure 5:**
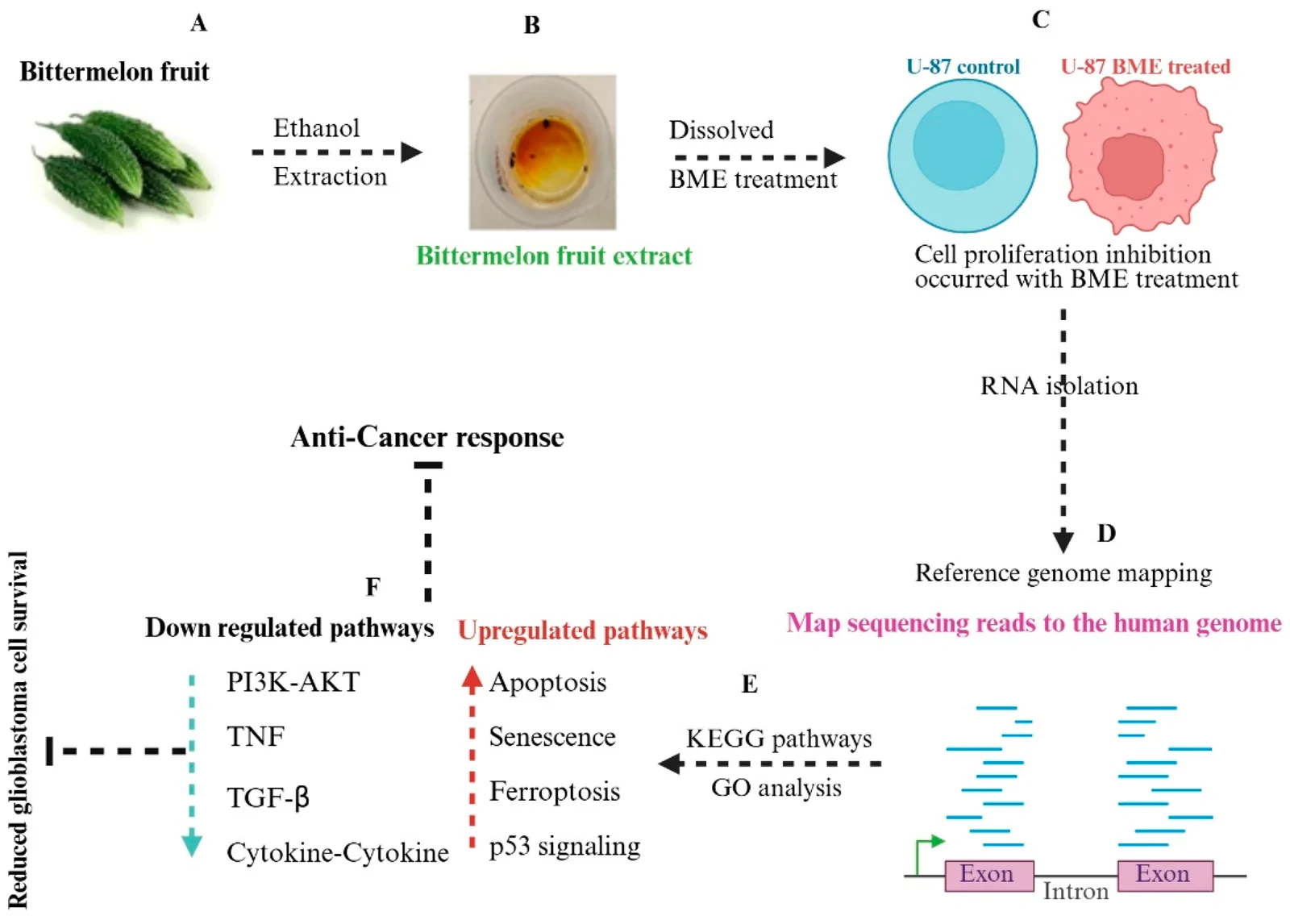
**Schematic overview of bitter melon extract (BME) treatment and its anticancer-associated transcriptomic response in U-87 glioblastoma cells.** (**A**) Bitter melon fruit was subjected to ethanol extraction to obtain the crude fruit extract. (**B**) The resulting BME was dissolved and prepared for cell treatment. (**C**) U-87 cells were treated with BME, resulting in inhibition of cell proliferation, followed by RNA isolation for transcriptomic analysis. (**D**) RNA-seq reads were mapped to the human reference genome to determine genome-wide gene expression changes. (**E**) Differentially expressed genes were subjected to KEGG pathway enrichment and Gene Ontology (GO) analyses. (**F**) Functional analysis revealed an anticancer-associated transcriptional response characterized by downregulation of pathways involved in PI3K–AKT, TNF, TGF-β, and cytokine–cytokine receptor signaling, together with upregulation of pathways associated with apoptosis, cellular senescence, ferroptosis, and p53 signaling. Collectively, these alterations suggest that BME suppresses pro-survival signaling while promoting cell-death and stress-response pathways in U-87 cells.

The metabolomic analysis of bitter melon fruit revealed a number of major bioactive compounds, the relative amounts of which are given in Table 2. This analysis found that several components of bitter melon fruit have previously exhibited anticancer activity in experimental models. Stigmasterol and β-sitosterol have been linked to cell-cycle arrest, the modulation of PI3K/Akt signaling, and the activation of apoptosis [14,15]. Caffeic and ferulic acids have also been reported to affect oxidative stress, mitochondrial function, and apoptosis-related signaling pathways [16,17]. Furthermore, α-linolenic, lauric, stearic, and palmitic acids have shown context-dependent antiproliferative or pro-apoptotic effects in certain cancer models [18,19,20,21], while azelaic and ascorbic acids may interfere with DNA synthesis and cellular redox homeostasis, respectively [22,23,24]. The metabolites identified in bitter melon fruit tissue provide phytochemical context for the observed activity of BME; however, direct chemical characterization and fractionation of the ethanolic extract will be required to determine which constituents contribute to its antiproliferative effects. In agreement with these results, BME significantly reduced the viability of U87 glioblastoma cells in a concentration- and time-dependent fashion (Figure 5), which is in line with earlier reports of its anticancer effects in other experimental models.

In order to examine the molecular mechanisms responsible for this growth-inhibitory effect, we then investigated the key signaling pathways and the genes that were differentially expressed after treatment with BME.

Transcriptome analysis showed that bitter melon extract (BME) slows U87 glioblastoma cell growth by affecting several signaling pathways at once. These include pathways for stress responses, apoptosis, senescence, inflammation, metabolism, and growth factor signaling. Rather than acting on a single pathway, BME caused broad changes in gene expression, activating p53-related stress responses while reducing inflammatory cytokine signaling, PI3K-AKT signaling, extracellular matrix remodeling, immune pathways, and lipid metabolism. These combined changes help explain how BME stops cell growth.

A major response to BME treatment was the activation of the p53 signaling pathway, as demonstrated by increased expression of GADD45A (1.15-fold), PTEN (1.01-fold), IGFBP3 (1.15-fold), SESN2 (1.25-fold), and SESN3 (1.78-fold). These genes regulate DNA damage responses, oxidative stress, cell-cycle arrest, and apoptosis, suggesting that BME induces genotoxic and oxidative stress sufficient to activate growth-inhibitory checkpoint pathways [29,30,31]. Consistent, upregulation of ATF4 (1.32-fold), DDIT3 (CHOP) (1.8-fold), FOS (3.5-fold), and JUN (1.17-fold) further indicates activation of endoplasmic reticulum stress and MAPK/AP-1 signaling, promoting stress-induced apoptosis [32,33]. Although BCL2A1 (1.3-fold) was also increased, this likely represents a compensatory survival response frequently observed in cancer cells exposed to cytotoxic stress.

BME also activated several senescence-associated pathways, including increased expression of TGFB2 (1.22-fold), GADD45A (1.15-fold), PTEN (1.01-fold), IGFBP3 (1.15-fold), IL1A (2.37-fold), CXCL8 (1.59-fold), SQSTM1 (1.27-fold), ZFP36L1 (1.29- fold), ZFP36L2 (1.14-fold), and SIRT1 (1.16-fold). These genes are associated with DNA damage signaling, oxidative stress adaptation, autophagy, and inflammatory senescence-associated secretory phenotype (SASP) components [34,35,36]. Although CDK1 (1.28-fold), CCNA2 (1.07-fold), and MYC (2.09-fold) were also upregulated, these genes may reflect compensatory cell-cycle responses during checkpoint activation rather than sustained proliferation. Together, these findings suggest that BME promotes a senescence-associated stress program that complements apoptotic signaling to inhibit glioblastoma cell growth.

Oxidative stress was further supported by upregulation of the ferroptosis-associated genes SLC7A11 (2.2-fold), SLC3A2 (1.71-fold), GCLC (1.04-fold), and HMOX1 (1.89-fold). These genes regulate cystine transport, glutathione biosynthesis, iron metabolism, and redox homeostasis, indicating activation of antioxidant defense mechanisms in response to elevated reactive oxygen species [37,38,39,40]. While these transcriptional changes alone do not confirm ferroptosis, they demonstrate substantial oxidative stress following BME treatment.

In contrast, BME markedly suppressed multiple oncogenic signaling pathways. Downregulation of IL7R (−1.6-fold), IL2RB (−1.87-fold), IL21R (−1.23-fold), CCL2 (−3.15-fold), CXCL1 (−1.0-fold), CXCL5 (−1.53-fold), CXCL16 (−2.37-fold), IL11 (−1.37-fold), IL33 (−1.08-fold), and CSF1R (−1.2-fold) suggests attenuation of cytokine-mediated inflammatory signaling that supports tumor growth and immune modulation [41,42,43]. Similarly, reduced expression of TRAF1 (−1.03-fold), VCAM1 (−3.24-fold), CCL2 (−3.15-fold), and CXCL1 (−1.0-fold) indicates suppression of the pro-inflammatory branch of TNF signaling [44,45,46], thereby limiting NF-κB-dependent survival signaling, chemokine production, and tumor-supportive microenvironment formation.

The PI3K–AKT signaling pathway, one of the most frequently activated pathways in glioblastoma, was also significantly suppressed. Reduced expression of PDGFRA (−4.28-fold), HGF (−1.48-fold), KDR (−1.29-fold), CSF1R (−1.2-fold), PRLR (−1.51-fold), PIK3R6 (−1.24-fold), and FGF7 (−1.76-fold) indicates decreased growth factor-mediated activation of PI3K–AKT signaling [47,48], whereas downregulation of COL1A1 (−1.86-fold), COL1A2 (−2.38-fold), COL4A3 (−1.17-fold), LAMC2 (−1.07-fold), and THBS4 (−1.36-fold) suggests impaired extracellular matrix remodeling and reduced invasive potential [49]. Furthermore, decreased ID1 (−1.01-fold), ID2 (−1.04-fold), and ID3 (−1.15-fold) expression indicates modulation of TGF-β/BMP-associated transcriptional programs involved in proliferation, stemness, and migration [50].

BME also attenuated innate immune and inflammatory signaling through reduced expression of IKBKE (−1.8-fold), AIM2 (−2.17-fold), STING1 (−2.78-fold), GBP1 (−1-fold), GBP2 (−1.334-fold), GBP5 (−1.36-fold), OAS1 (−1.38-fold), OAS2 (−1.09-fold), and NLRP12 (−1.55-fold), indicating suppression of inflammasome- and interferon-associated pathways [51,52]. In addition, downregulation of ACSL5 (−1.42-fold), ACSBG2 (−1.19-fold), OLAH (−1.472-fold), and HSD17B8 (−1.168-fold) suggests disruption of fatty acid metabolism, reducing lipid availability required for membrane biosynthesis and rapid tumor cell proliferation [53,54].

Collectively, these findings demonstrate that BME inhibits glioblastoma cell growth through a multifaceted mechanism involving activation of p53-dependent stress responses, apoptosis, senescence-associated pathways, and oxidative stress while suppressing inflammatory cytokine signaling, PI3K–AKT signaling, TGF-β/BMP-associated transcriptional programs, innate immune pathways, extracellular matrix remodeling, and lipid metabolism (Figure 5). This coordinated transcriptomic reprogramming highlights BME’s potential as a multi-target therapeutic candidate for glioblastoma and provides a strong molecular basis for its anticancer activity.

A primary limitation of this study is that the proposed molecular mechanisms are inferred mainly from transcriptomic profiling and bioinformatic pathway enrichment analyses. While these results offer valuable insights into the potential anticancer effects of bitter melon extract (BME), changes in mRNA abundance may not directly correspond to alterations in protein expression or functional activity. Consequently, further validation of the identified pathways and key regulatory genes is required through complementary methods such as pathway-specific functional experiments. Additional research using appropriate in vitro and in vivo models is also necessary to confirm the biological and therapeutic relevance of these transcriptome-based findings.

## 4. Materials and Methods

### 4.1. The Plant Material and the Preparation of the Bitter Melon Extract

The bitter melon (*Momordica charantia* L.) fruits was purchased from local farmers’ market. The seeds were removed from the fruits, and both the parts were dried at room temperature before grinding into a fine powder. To prepare the bitter melon extract (BME) to use in the cell-based assays, 40 g of the dried fruit powder was suspended in 500 mL of 70% (*v*/*v*) ethanol and stirred at 65 °C overnight. The mixture was then filtered under vacuum to eliminate the insoluble material, and the resulting filtrate was concentrated overnight in a vacuum oven at 50 °C to remove the solvent. The dried extract was weighed, placed into a sterile tube, and stored in the freezer at −20 °C until it was required for the cell-based experiments.

### 4.2. Metabolite Profiling of Bitter Melon Fruit Tissue by Means of GC–MS

A portion of fresh bitter melon (*Momordica charantia*) fruit tissue from the same biological material used to prepare the 70% ethanol extract (BME) was reserved for metabolite profiling by gas chromatography–mass spectrometry (GC-MS). The analysis was performed at the Roy J. Carver Biotechnology Center, University of Illinois Urbana-Champaign, USA, to characterize the metabolite composition of the starting fruit material. Metabolites were extracted from homogenized fresh fruit tissue, and the resulting supernatant was dried prior to derivatization. The dried extracts were treated with methoxyamine hydrochloride in pyridine, followed by trimethylsilylation using N-methyl-N-(trimethylsilyl)trifluoroacetamide (MSTFA). Hentriacontanoic acid (C31) was used as an internal standard for normalization. Because BME was prepared using 70% ethanol to recover soluble phytochemicals across a range of polarities, the GC-MS profile of fresh fruit tissue was intended to provide complementary information on the phytochemical composition of the source material rather than a quantitative representation of the final extract. These metabolite data were used to provide chemical context for interpreting the cellular and transcriptomic responses of U87 MG glioblastoma cells following BME treatment.

The derivatized samples were investigated with a gas chromatograph of the Agilent 7890 model together with an Agilent 5975 mass-selective detector and a ZB-5MS capillary column. Helium was used as the carrier gas, and mass spectra were obtained by means of electron-impact ionization. The chromatographic peaks were dealt with by means of the Automated Mass Spectral Deconvolution and Identification System (AMDIS). The metabolites were identified by comparing their mass spectra and retention times with those in the NIST, Wiley and in-house spectral libraries. The abundance of the metabolites was adjusted in relation to the internal standard and the fresh tissue weight and is given as relative concentration per gram of fresh weight.

### 4.3. Treatments

To prepare the stock solution for use in cell-based treatments, a portion of the stored bitter melon extract (BME) was taken. We transferred about 510 mg of BME into a 2 mL microcentrifuge tube that had already been weighed. The weight of the empty tube was 1.097 g, and the weight of the tube with the extract in it was 1.607 g; this amount corresponds to 510 mg of BME. The extract was first dissolved in 200 µL of ethanol and then made up to a final volume of approximately 1.815 mL using cell culture medium, giving a BME stock concentration of 280 mg/mL. The solution was then thoroughly homogenized, and 1.5 mL of it was transferred to a sterile 2 mL tube in order to obtain the required BME treatment concentrations.

In the case of the cell-based experiments, we diluted the BME stock using culture medium in order to achieve the required treatment concentrations. When we used the highest BME concentration tested (840 µg/mL), the final ethanol concentration was about 0.033% (*v*/*v*). To rule out any possible solvent effect on cell viability, we added an equivalent concentration of ethanol to the vehicle-control cells in the culture medium.

### 4.4. Cell Culture

Human glioblastoma cell line (U87 MG) was purchased from the American Type Culture Collection (ATCC, Manassas, VA, USA). Cells were grown in T75 or T175 culture flasks (Greiner, Monroe, NC, USA) in high-glucose Dulbecco’s Modified Eagle Medium (ATCC, Manassas, VA, USA) supplemented with 10% fetal bovine serum (Atlas Biologicals, Fort Collins, CO, USA) and 1x antibacterial/antimycotic solution (Gibco, Grand Island, NY, USA). The cells were maintained in a humidified incubator at 37 °C and in a gas mixture of 5% CO_2_ and 95% air.

### 4.5. Cell Viability Assay

The effects of increasing concentrations of BME on U87 MG cell viability were assessed. In total, 5 × 10^3^ cells were seeded into each well of a 96-well microplate in 100 μL of medium and incubated overnight. Cells were treated with 0 μg/mL, 280 μg/mL, 420 μg/mL, 560 μg/mL, 700 μg/mL and 840 μg/mL BME and ethanol (vehicle control) for 24, 48, and 72 h. Cell viability was determined using the Cell Counting Kit-8 (CCK-8; Dojindo Molecular Technologies, Inc., Kumamoto, Japan) assay. Briefly, after treatment, 10 μL of CCK-8 solution was added to each well containing 100 μL of treatment medium. The plates were incubated for 4 h. Subsequently, the optical density at 450 nm was read using the SpectraMax iD3 Multi-Mode Microplate Reader (Molecular Devices, San Jose, CA, USA). The cytotoxicity of BME was assessed by determining the IC_50_ from the dose–response curve obtained using a cell viability assay at 24 h.

### 4.6. RNA Isolation and Library Preparation

The effect of BME treatment on global gene expression in U87-MG cells was assessed using RNA-seq. Three replicates of each of two experimental conditions were set up: negative control (vehicle-treated cells) and treated cells. Cells were cultured in 60 mm × 15 mm cell culture dishes (Wuxi NEST Biotechnology Co., Ltd., Wuxi, Jiangsu, China) until 80–90% confluence and treated with 659 μg/mL BME. After 24 h of incubation, RNA was isolated using the RNeasy Plus Mini Kit (QIAGEN, Germantown, MD, USA) and stored at −20 °C until sequencing.

### 4.7. RNA-Seq Analysis

Sequencing reads were processed by trimming adapters and removing low-quality bases (Phred score < 30) using Trimmomatic v0.39 [55]. The filtered reads were then aligned to the human reference genome (GRCh38.p13) using the STAR RNA-Seq aligner v2.7.11a [56], generating BAM alignment files. Read quantification was performed with the HTSeq R package (version 2.0.3) [57] using genome annotations in GFF format. DEG analysis was conducted using DESeq2 [58], considering genes with |log_2_FC| ≥ 1 and FDR ≤ 0.05.

### 4.8. Pathway Enrichment Analysis

Kyoto Encyclopedia of Genes and Genomes (KEGG) pathway enrichment analysis was carried out using KOBAS (http://kobas.cbi.pku.edu.cn; accessed on [8 June 2026]) to identify biological pathways associated with the DEGs. KEGG pathway annotations were attained through the KEGG database (https://www.kegg.jp/; accessed on [8 June 2026]) [59]. These analyses were employed to characterize the functional implications of treatment-associated transcriptional changes in U87 MG glioblastoma cells.

Functional enrichment analysis was performed to investigate the biological functions and molecular pathways associated with DEGs identified through RNA-seq analysis. Gene Ontology (GO) enrichment analysis was conducted using gprofiler (https://biit.cs.ut.ee/gprofiler; accessed on [8 June 2026]) to classify the DEGs into three major functional categories: biological processes (BPs), molecular functions (MFs), and cellular components (CCs) [60].

### 4.9. Statistical Analysis

All statistical analyses were conducted using GraphPad Prism v10.4.0.621 for Windows (GraphPad Software, Boston, MA, USA; www.graphpad.com; accessed on 1 December 2024). A *p*-value of <0.05 was considered statistically significant.

## Figures and Tables

**Table 1:** A classification of the metabolites identified in the bitter melon fruit. We grouped the metabolites into 11 major chemical and metabolic categories based on their structural features and known biochemical functions. Representative compounds for each category are shown.

S. No.	Major Class	Representative Metabolite
**1**	**Sugars and oligosaccharides**	Glucose, fructose, galactose, sucrose, raffinose, maltose, melibiose, arabinose, ribose, xylose, xylulose, glucose, sedoheptulose
**2**	**Sugar acids and lactones**	Galactonic acid, galactonic acid-1,4-lactone, gulonic acid, gluconic acid, gluconic acid lactones, galactaric acid, ribonic acid, threonic acid, erythronic acid
**3**	**Sugar alcohols/polyols**	Inositol, glycerol, arabitol, erythritol, xylitol, 1,2-propanediol
**4**	**Phosphorylated carbohydrates**	Glucose-6-P, fructose-6-P, mannose-6-P, inositol-P, gluconic acid-6-P, 3-phosphoglycerate
**5**	**Amino acids and derivatives**	Alanine, aspartic acid, glycine, β-alanine, GABA, pyroglutamic acid
**6**	**Amines/polyamines and nitrogen metabolites**	Putrescine, butylamine, ethanolamine, 2-aminoethylphosphate, urea
**7**	**Organic acids/central carbon metabolites**	Citric acid, malic acid, succinic acid, fumaric acid, α-ketoglutaric acid, pyruvic acid, lactic acid, oxalic acid
**8**	**Fatty acids and lipid-related metabolites**	Palmitic, stearic, oleic, linoleic, linolenic, decanoic, dodecanoic, tetradecanoic, eicosanoic and docosanoic acids; monoacylglycerols
**9**	**Phenolic/aromatic acids**	Caffeic acid, ferulic acid, p-hydroxybenzoic acid, benzoic acid
**10**	**Sterols, vitamins and antioxidants**	Sitosterol, stigmasterol, α-tocopherol, ascorbic acid, dehydroascorbic acid
**11**	**Nucleosides and miscellaneous metabolites**	Adenosine, uridine, phosphoric acid, hydroxylamine, triethanolamine, monomethylphosphate

**Table 2:** Cancer-associated bioactive metabolites identified in bitter melon fruit. The table presents selected metabolites with previously reported antiproliferative, pro-apoptotic, anti-invasive, anti-metastatic, or other cancer-related activities, as well as their relative concentrations normalized to fresh weight (RC FW^−1^) and the supporting literature references. The reported activities are primarily derived from preclinical studies and do not necessarily indicate clinically established anticancer efficacy.

S. No.	Active Metabolite	RC Fw-g-1	Reported Cancer Activity	Reference
**1**	Stigmasterol	100.1	Antiproliferative, pro-apoptotic, and anti-invasive effects reported in preclinical cancer models	[14]
**2**	Sitosterol (β-sitosterol)	19.0	Antiproliferative, apoptosis-inducing, and cell-cycle regulatory activities	[15]
**3**	Caffeic acid	23.9	Antiproliferative and pro-apoptotic activities reported in experimental cancer models	[16]
**4**	Ferulic acid	5.5	Antiproliferative, pro-apoptotic, and antiangiogenic effects	[17]
**5**	9,12,15-Octadecatrienoic acid (α-linolenic acid)	140.2	Concentration-dependent inhibition of cancer cell viability; apoptosis	[18]
**6**	Dodecanoic acid (lauric acid)	15.2	Suppresses proliferation and induces apoptosis	[19]
**7**	Stearic acid	908.1	Preferential induction of apoptosis; inhibition of proliferation/invasion reported	[20]
**8**	Palmitic acid	1188.8	Antiproliferative and pro-apoptotic effects reported in several cancer models	[21]
**9**	Azelaic acid	11.0	Inhibits proliferation and DNA synthesis; antineoplastic activity in melanoma models	[22]
**10**	Ascorbic acid (vitamin C)	9.2	Pharmacologic-dose anticancer activity under investigation; cytotoxicity in preclinical models	[23]
**11**	Dehydroascorbic acid	80.8	Cancer cell redox disruption and cytotoxicity reported; proposed mediator of high-dose vitamin-C effects	[24]

## Data Availability

The raw paired-end Illumina RNA-sequencing reads generated in the current study are available in the Sequence Read Archive (SRA) at NCBI under the Bioproject accession numbers PRJNA1528488.

## Supplementary Materials

The following supporting information can be downloaded at: https://www.mdpi.com/article/10.3390/molecules31203714/s1.
